# Age- and sex-specific normative values for muscle mass parameters in 18,625 Brazilian adults

**DOI:** 10.3389/fpubh.2023.1287994

**Published:** 2024-01-03

**Authors:** Hélio José Coelho-Júnior, Fillipi Lopes Marques, Caio Victor Sousa, Emanuele Marzetti, Samuel da Silva Aguiar

**Affiliations:** ^1^Department of Geriatrics, Orthopedics, and Rheumatology, Università Cattolica del Sacro Cuore, Rome, Italy; ^2^Department of Physical Education, University Center–UDF, Brasília, Brazil; ^3^Health and Human Sciences, Loyola Marymount University, Los Angeles, CA, United States; ^4^Fondazione Policlinico Universitario “A. Gemelli” IRCCS, Rome, Italy

**Keywords:** sarcopenia, frailty, anorexia, muscle atrophy, weight loss, aged

## Abstract

**Background:**

The present study aimed to provide age- and sex-specific normative values for muscle mass parameters in Brazilian adults.

**Methods:**

Data pertaining to Brazilian adults (18+ years) who attended a nutritional clinical between January 2018 and July 2022 were analyzed. Muscle mass parameters were assessed using a bioimpedance digital scale (InBody 230, GBC BioMed NZ). Assessments were conducted under standard conditions, with participants refraining from physical exercise for 96 h and from eating or drinking (including water) for 8 h before evaluations.

**Results:**

A total of 18,625 Brazilian adults were analyzed. Normative values for absolute and relative (height, m^2^) muscle mass and appendicular muscle mass (ASM) were calculated. In addition, specific age-related changes in muscle mass parameters were observed. In women, muscle mass peaked between the ages of 40–49 before gradually declining at an average rate of 5.7% per decade from the sixth decade of life onwards. ASM reached its peak earlier, during the third decade of life, and started to decline later, from 50 to 59 years. In contrast, absolute and ASM peaked at 40–49 years and declined from the sixth decade of life in men. Both sexes displayed a slightly greater decline in ASM than in muscle mass (13 vs. 12%).

**Conclusions:**

The present study provides normative values for absolute and relative muscle mass and ASM in Brazilian adults. Furthermore, important specific age-related changes in muscle mass parameters were observed. These data have public health implications and might serve as a reference tool to guide health professionals.

## 1 Introduction

The skeletal muscle is an active endocrine tissue that serves as a source of protein, with key influences on glucose and energy metabolism, and a major role in mobility (1, 2). Muscle mass progressively declines with age, and numerous studies have observed that this scenario is of public health concern, given its independent associations with negative outcomes, including diminished physical performance, mobility loss, osteoarthritis, dementia, and death (3–6). Furthermore, muscle atrophy contributes to significant impairments in neuromuscular function (7, 8), mainly with the development of muscle strength and power, thereby promoting sarcopenia. As such, the monitoring of muscle mass across the lifespan is vital to public health programs that aim to understand patients' health status, early identify people at risk of negative conditions, and properly provide therapeutic interventions.

Body-imaging techniques [i.e., Dual Energy X-ray Absorptiometry (DEXA), computed tomography (CT), and magnetic resonance imaging (MRI)] are recognized as gold standard methods to estimate muscle mass (9, 10). However, the utilization of these devices is hampered mainly in primary care settings due to their high costs, lack of portability, and the need for specialized personnel to operate the equipment and interpret results (9, 10).

Bioelectrical impedance analysis (BIA) is a simple, non-invasive, and inexpensive method that estimates muscle mass by calculating tissue conductivity (11). It has been suggested as a good portable alternative method to body-imaging techniques by providing reliable results, especially in population-based studies, serving as a valid instrument to be used in public health programs to identify people at risk (11). In fact, significant correlations have been observed between BIA and MRI (7) as well as DEXA (12, 13). In addition, normative values have been established for different populations (8, 14–17).

Notably, most studies reporting normative data examined samples composed of European and North American people (8, 14–17), whereas studies in South America are scarce. This deserves concern since older adults represent more than 10% of the South American population and it is estimated that this proportion will exceed 25% by 2050 (18). Brazil is one of the top three “oldest” countries in Latin America, with an old population of more than 30 million people (18). Moreover, the lack of normative values for muscle mass parameters in the Brazilian population still hampers the accurate diagnosis of sarcopenia in this population, which is based on non-specific cutoff points (19).

Based on these premises, the present study analyzed a large database to provide age- and sex-specific normative values for muscle mass parameters of Brazilian adults.

## 2 Materials and methods

### 2.1 Study participants and data collection

This is a retrospective study that examined a comprehensive cohort of patients who attended a Nutritional Clinic, in Brasilia, Brazil. The clinic has two locations, both in the city center, opens six days a week, integrates the work and expertise of health professionals from different backgrounds, including nutritionists, exercise physiologists, psychologists, physicians, and surgeons, and has been operating for more than 15 years. Data from patients aged 18+ years, who had attended the clinic between January 2018 and July 2022 were analyzed. People with incomplete data, water or electrolyte imbalances (e.g., edema and ascites), skin abnormalities (e.g., pachydermia secondary to hypothyroidism), abnormal body geometry (e.g., amputation and limb atrophy), and self-reported pregnancy were excluded from the analysis. We also excluded participants who did not follow testing recommendations described in subtopic 2.2, body composition. The study was conducted in accordance with the Declaration of Helsinki and was approved by the Ethics Committee of University Centre UDF (protocol #: 5.975.561). The manuscript was prepared in accordance with the Strengthening the Reporting of Observational Studies in Epidemiology (STROBE) guidelines for observational studies (20).

### 2.2 Body composition

Body height and mass were measured through a stadiometer and an analog medical scale, respectively. The body mass index (BMI) was then calculated as the ratio between body mass (kg) and the square of height (m^2^). Muscle mass parameters were estimated using a BIA scale (InBody 230, GBC BioMed NZ) (21, 22) under standard conditions (23). The InBody 230 BIA scale utilizes a hand-to-foot tetrapolar 8-point tactile electrodal bioelectrical impedance system to estimate body composition (22). Before testing, participants had their hands and feet cleaned with alcohol. The device was programmed according to test person's age, sex, and height by a staff member. During testing, volunteers remained standing, with feet positioned in four electrodes (two each), while the other four electrodes were held by the hands. Participants were not allowed to rest their arms along the torso during evaluation. Each measure takes ~30 s and involves two frequencies (20 kHz and 100 kHz) (22).

All evaluations occurred throughout the day (i.e., from 8 am to 5 pm). During scheduling, participants were instructed to refrain from physical exercise for 96 h and from eating or drinking (including water) for 8 h before evaluations. They were also invited to urinate at least 30 min before evaluation. Upon arriving at the clinic, patients were asked if they had followed the recommendations for testing.

Appendicular muscle mass (ASM) was calculated as the sum of the muscle masses of upper (i.e., left and right arms) and lower (i.e., left and right legs) limbs. Skeletal muscle indexes I and II were calculated as follows (10).

a) SMI I: absolute muscle mass/height^2^.
b) SMI II: ASM/height^2^.

### 2.3 Statistical analysis

Characteristics of study participants according to sex and age groups are summarized as means ± standard deviation (SD) or percent (%). One-way ANOVA, with Bonferroni's *post-hoc* test, was used to compare participant characteristics among age groups in the whole sample and in men and women separately. The percentages of differences were determined by calculating the variances between the values of the 18–29 group and the other age groups. Then, the “rule of three” method was applied to calculate the proportion of variations, using the 18–29 group as the reference, as follows:


$$c)X\%\;=\frac{\text{18–29 years value}{\;}^{*}100}{\text{Variances}\;(\text{18–29 years value – value of other age group})}$$

Pearson's correlations were run to explore the relationship between muscle mass parameters and age. For all tests, the level of significance was set at 5% (*P* < 0.05). All analyses were performed using the SPSS software version 23.0 (SPSS Inc., Chicago, IL). Smoothed percentile curves for absolute muscle power values in men and women were constructed using the lambda-mu-sigma (LMS) method (LMS Chart Maker Pro Version 2.54, Medical Research Council, London, UK), as described elsewhere (24).

## 3 Results

### 3.1 Participants characteristics

Data from 18,625 (10, 572 ♀, 8, 053♂) Brazilian adults were examined. The main characteristics of study participants are shown in Table 1. In women, BMI values increased significantly from the fourth to the sixth decade of life. Muscle mass increased significantly until 40–49 years and started to decrease from the fifth decade of life. However, statistical significance was only observed from the sixth decade of life. A different pattern was observed for ASM, which had an earlier peak, at 30–39 years, and then, significantly dropped from the fifth decade of life until 70–79 years. Most of these results were influenced by age-related declines in lower-limb muscle mass, given that it declined earlier and to a greater extent when compared to upper-limb muscles. SMI I, based on muscle mass, was significantly higher across all age groups, except 80+, when compared to 18–29 years. In contrast, SMI II, based on ASM, increased from 30–39 to 50–59 years and then significantly declined from the seventh decade of life.

**Table 1:** Characteristics of study participants (*n* = 18,625).

**Variables**	**Age groups (years)**						
**Women (*****n** =* **10,572)**	**18–29 (*****n** =* **1,544)**	**30–39 (*****n** =* **2,452)**	**40–49 (*****n** =* **3,101)**	**50–59 (*****n** =* **1,878)**	**60–69 (*****n** =* **1,127)**	**70–79 (*****n** =* **368)**	**80**+ **(*****n** =* **102)**
Age (years)	25.1 ± 3.1	34.6 ±2.9^a^	44.1 ± 2.8^ab^	54.2 ± 2.8^abc^	64.0 ± 3.0^abcd^	73.4 ± 2.5^abcde^	83.6 ± 3.7^abcdef^
BMI (kg/m^2^)	27.6 ± 5.5	27.6 ± 5.0	28.4 ± 4.7^ab^	28.7 ± 4.5^ab^	29.2 ± 4.0^abc^	30.4 ± 5.7^abcde^	30.4 ± 4.1^abcd^
Muscle mass (kg)	25.2 ± 3.0	25.7 ± 3.3^a^	25.6 ± 3.2^a^	25.0 ± 3.1	23.9 ± 2.7^abcd^	23.5 ± 2.9^abcd^	23.6 ± 2.6^abcd^
Right arm muscle mass (kg)	2.4 ± 0.3	2.5 ± 0.4^a^	2.5 ± 0.4^ab^	2.5 ± 0.3^abc^	2.4 ± 0.3^ac^	2.3 ± 0.3^bcd^	2.3 ± 0.3^bcd^
Left arm muscle mass (kg)	2.4 ± 0.3	2.5 ± 0.4^a^	2.5 ± 0.4^ab^	2.4 ± 0.3^a^	2.4 ± 0.3^bcd^	2.3 ± 0.3^bcd^	2.3 ± 0.3^bcd^
Right leg muscle mass (kg)	7.1 ± 0.9	7.2 ± 1.0^a^	7.1 ± 0.9^b^	6.8 ± 0.9^abc^	6.3 ± 0.8^abcd^	6.1 ± 0.8^abcde^	6.2 ±1.1^abcd^
Left leg muscle mass (kg)	7.0 ± 0.9	7.1 ± 0.9^a^	7.0 ± 0.9^b^	6.7 ± 0.9^bc^	6.3 ± 0.8^abcd^	6.0 ± 0.7^abcde^	6.1 ± 0.9^abcd^
Appendicular muscle mass (kg)	19.0 ± 2.4	19.3 ± 2.7^a^	19.2 ± 2.5	18.5 ± 2.4^abc^	17.5 ± 2.2^abcd^	16.9 ± 2.0^abcde^	17.0 ± 2.3^abcd^
SMI I (kg/m^2^)	9.2 ± 0.9	9.4 ± 0.9^a^	9.5 ± 0.9^ab^	9.5 ± 0.9^ab^	9.4 ± 0.8^ac^	9.5 ± 1.2^a^	9.5 ± 0.9
SMI II (kg/m^2^)	7.0 ± 0.6	7.1 ± 0.7^a^	7.1 ± 0.6^a^	7.0 ± 0.6^ac^	6.9 ± 0.6^bcd^	6.8 ± 0.7^abcd^	6.8 ± 0.7^bcd^
**Men (*****n** =* **8,053)**	**18–29 (*****n** =* **1,793)**	**30–39 (*****n** =* **1,885)**	**40–49 (*****n** =* **1,911)**	**50–59 (*****n** =* **1,077)**	**60–69 (*****n** =* **755)**	**70–79 (*****n** =* **410)**	**80**+ **(*****n** =* **222)**
Age (years)	24.0 ± 3.3	34.8 ± 2.8^a^	44.2 ± 2.8^ab^	54.0 ± 2.8^abc^	63.8 ± 2.8^abcd^	73.5 ± 2.9^abcde^	85.7 ± 4.3^abcdef^
BMI (kg/m^2^)	25.5 ± 4.9	28.4 ± 4.8^a^	29.1 ± 5.0^ab^	28.6 ± 4.7^ac^	28.8 ± 4.2^a^	26.9 ± 4.1^abcde^	26.7 ± 3.9^abcde^
Muscle mass (kg)	34.7 ± 5.2	37.2 ± 5.4^a^	37.3 ± 5.2^ac^	35.2 ± 4.8^bc^	33.3 ± 4.9^abcd^	30.6 ± 4.2^abcde^	27.6 ± 30.8^abcdef^
Right arm muscle mass (kg)	3.5 ±0.6	3.9 ± 0.6^a^	3.9 ± 0.6^a^	3.7 ± 0.5^abc^	3.4 ±0.5^bcd^	3.1 ± 0.5^abcde^	2.7 ± 0.4^abcdef^
Left arm muscle mass (kg)	3.5 ± 0.7	3.8 ± 0.6^a^	3.9 ±0.6^a^	3.6 ± 0.6^bc^	3.4 ±0.5^abcd^	3.1 ± 0.5^abcde^	2.7 ± 0.4^abcdef^
Right leg muscle mass (kg)	9.5 ± 1.3	9.9 ± 1.3^a^	9.9 ± 1.3^a^	9.4 ± 1.3^abc^	8.9 ± 1.4^bcd^	8.2 ± 1.2^abcde^	7.5 ± 1.2^abcdef^
Left leg muscle mass (kg)	9.4 ± 1.3	9.9 ± 1.3^a^	9.8 ± 1.3^a^	9.3 ± 1.3^bc^	8.9 ± 1.3^abcd^	8.1 ± 1.2^abcde^	7.4 ± 1.0^abcdef^
Appendicular muscle mass (kg)	26.1 ± 3.8	27.6 ± 3.9^a^	27.6 ± 3.7^a^	26.2 ± 3.7^bc^	24.8 ± 3.8^abcd^	22.7 ± 3.3^abcde^	20.4 ± 3.0^abcdef^
SMI I (kg/m^2^)	11.0 ± 1.2	11.8 ± 1.1^a^	11.9 ± 1.2^a^	11.5 ± 1.1^abc^	11.2 ± 1.1^bcd^	10.6 ± 1.0^abcde^	10.0 ± 1.0^abcdef^
SMI II (kg/m^2^)	8.3 ± 0.8	8.8 ± 0.7^a^	8.8 ± 0.8^a^	8.6 ± 0.7^abc^	8.3 ± 0.8^bcd^	7.8 ± 0.7^abcde^	7.3 ± 0.7^abcdef^

BMI, Body mass index; SMI, Skeletal muscle index. ^*a*^P < 0.05 vs. 18–29. ^*b*^P < 0.05 vs. 30–39. ^*c*^P < 0.05 vs. 40–49. ^*d*^P < 0.05 vs. 50–59. ^*e*^P < 0.05 vs. 60–69. ^*f*^P < 0.05 70–79.

In men, BMI increased significantly from the third decade of life until 40–49 years, and then declined from 60 to 69 years until 80+. Men in the 30–39 and 40–49 years age groups had more muscle mass than those aged 18–29 years. A significant and linear decline in muscle mass was observed from 60 to 69 years until 80+ years. A similar pattern of age-related changes was observed in ASM. In this case, upper- and lower-limb seemed to have influenced changes in ASM. SMI I and II increased until 50–59 years followed by a continuous decline from the seventh decade of life.

### 3.2 Muscle mass across age groups

Mean and percent differences in muscle mass parameters across ages compared with the 18–29 years group are shown in Table 2. In women, muscle mass reached a peak at 40–49 years. It started to decline continuously from the fifth decade of life, with slight changes observed in those 50–59 years (−0.7%) and considerable reductions noted in the subsequent age groups. During old age, muscle mass declined by 4.7% in those 60–69 years and 6.3% in those 70–79 and 80+ years. A mean decline rate of 5.7% per decade was observed from the sixth decade of life. A similar pattern of age-related changes was observed for ASM. However, greater reductions were noted during old age, with a mean rate of decline of 9.4% per decade. Higher SMI I was observed across all age groups in comparison to those 18–29 years. A mean SMI I of ~2.0% was observed. These values were slightly lower during old age (mea*n* = 1.7%). In contrast, SMI II increased until the fourth decade of life, did not change in those 50–59 years and 60–69 years, and declined at a rate of 1.4% per decade from the seventh decade of life.

**Table 2:** Mean and percent differences in muscle mass parameters relative to the 18–29 years group.

**Variables**	**Age groups**					
**Women**	**30–39 (*****n** =* **2,452)**	**40–49 (*****n** =* **3,101)**	**50–59 (*****n** =* **1,878)**	**60–69 (*****n** =* **1,127)**	**70–79 (*****n** =* **368)**	**80**+ **(*****n** =* **102)**
Muscle mass (kg)	0.4 (+1.5%)	0.4 (+1.5%)	−0.2 (−0.7%)	−1.2 (−4.7%)	−1.6 (−6.3%)	−1.6 (−6.3%)
Appendicular muscle mass (kg)	0.3 (+1.5%)	0.2 (+1.0%)	−0.4 (−2.0%)	−1.4 (−7.3%)	−2.0 (−10.5%)	−2.0 (−10.5%)
Skeletal muscle index I (kg/m^2^)	0.1 (+1.0%)	0.3 (+3.2%)	0.2 (+2.1%)	0.1 (+1.0%)	0.2 (+2.1%)	0.2 (+2.1%)
Skeletal muscle index II (kg/m^2^)	0.1 (+1.4%)	0.1 (+1.4%)	0.0 (0.0%)	0.0 (0.0%)	−0.1 (−1.4%)	−0.1 (−1.4%)
**Men**	**30–39 (*****n** =* **1,885)**	**40–49 (*****n** =* **1,911)**	**50–59 (*****n** =* **1,077)**	**60–69 (*****n** =* **755)**	**70–79 (*****n** =* **410)**	**80**+ **(*****n** =* **222)**
Muscle mass (kg)	2.5 (+7.2%)	2.5 (+7.2%)	0.4 (+1.1%)	−1.4 (−4.0%)	−4.1 (−11.8%)	−7.0 (−20.1%)
Appendicular muscle mass (kg)	1.5 (+5.7%)	1.5 (+5.7%)	−0.1 (−0.3%)	−1.2 (−4.5%)	−3.3 (−12.6%)	−5.6 (−21.4%)
Skeletal muscle index I (kg/m^2^)	0.7 (+6.3%)	0.8 (+7.2%)	0.5 (+4.5%)	0.1 (+0.9%)	−0.4 (−3.6%)	−1.0 (−0.9%)
Skeletal muscle index II (kg/m^2^)	0.4 (+4.8%)	0.5 (+6.0%)	0.3 (+3.6%)	0.0 (0.0%)	−0.4 (−4.8%)	−0.9 (−10.8%)

In men, muscle mass reached a peak at 40–49 years. Men 50–59 years still had more muscle mass than those 18–29 years, although values were only slightly greater (1.1%). Muscle mass started to decline at the sixty decade of life, with older men 60–69 displaying 4.0% less muscle mass. Men 80+ had ~20% less muscle mass than the reference group. Muscle mass declined at a mean rate of 12.0% per decade from 60–69 up to 80+. ASM reached a peak at 30–39 years, remained higher in men 40–49 years, and started to decline from the fifth decade of life. In old age, ASM had declined 4.5% in those 60–69 years, 12.6% in those 70–79 years, and 21.4% in those 80+. A mean decline rate of ~13% was observed from the sixth decade of life. A similar pattern was observed for SMI I and II, given that both measures increased until approximately the sixth decade of life and then declined. Notably, SMI II displayed larger age-related changes in comparison to SMI I.

### 3.3 Associations between age and muscle mass parameters

**Figure 1:**
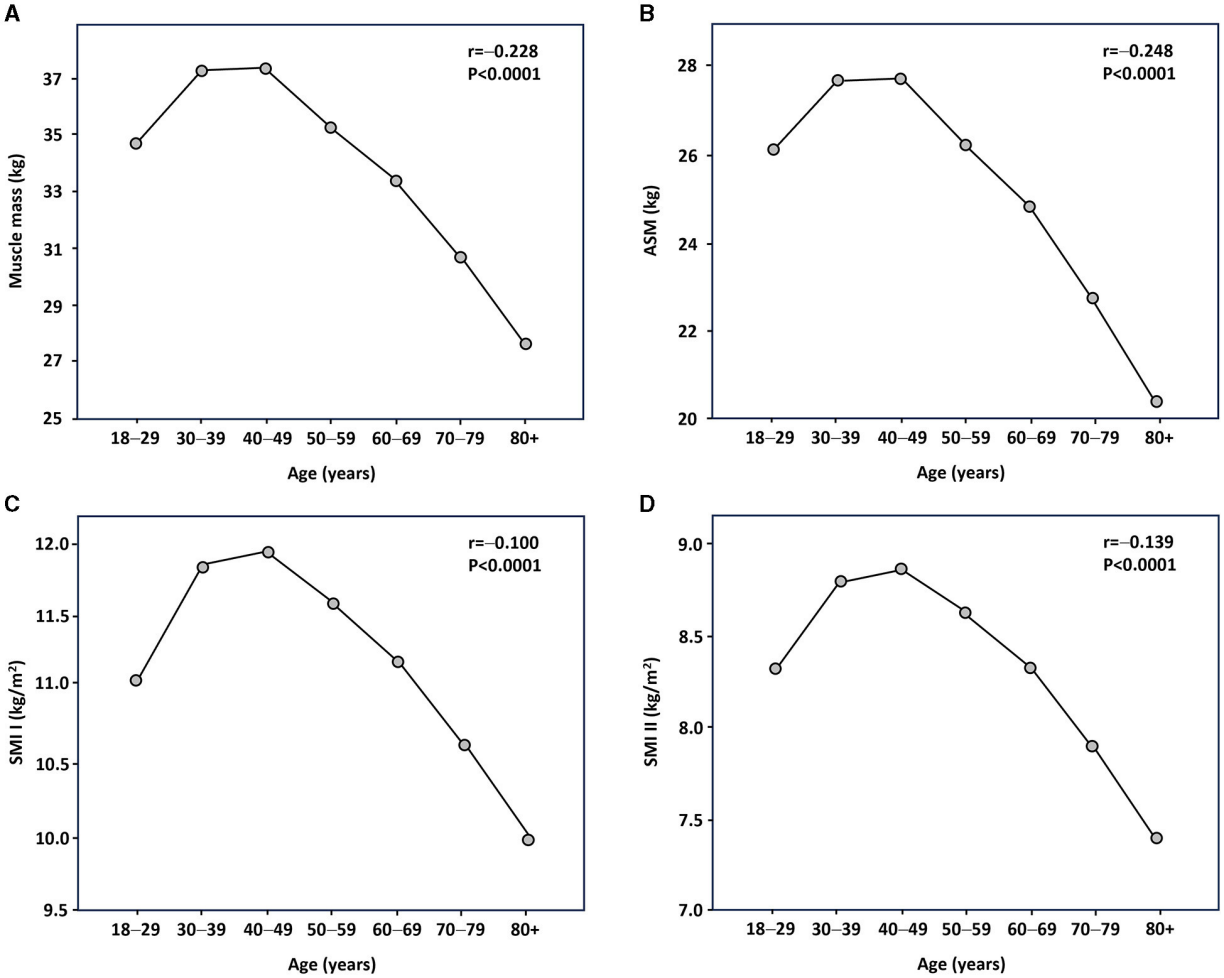
Relationship between age and muscle mass parameters in female participants as assessed by Pearson's statistics. **(A)** Muscle mass; **(B)** appendicular skeletal muscle (ASM); **(C)** skeletal muscle index (SMI) I; and **(D)** SMI II.

**Figure 2:**
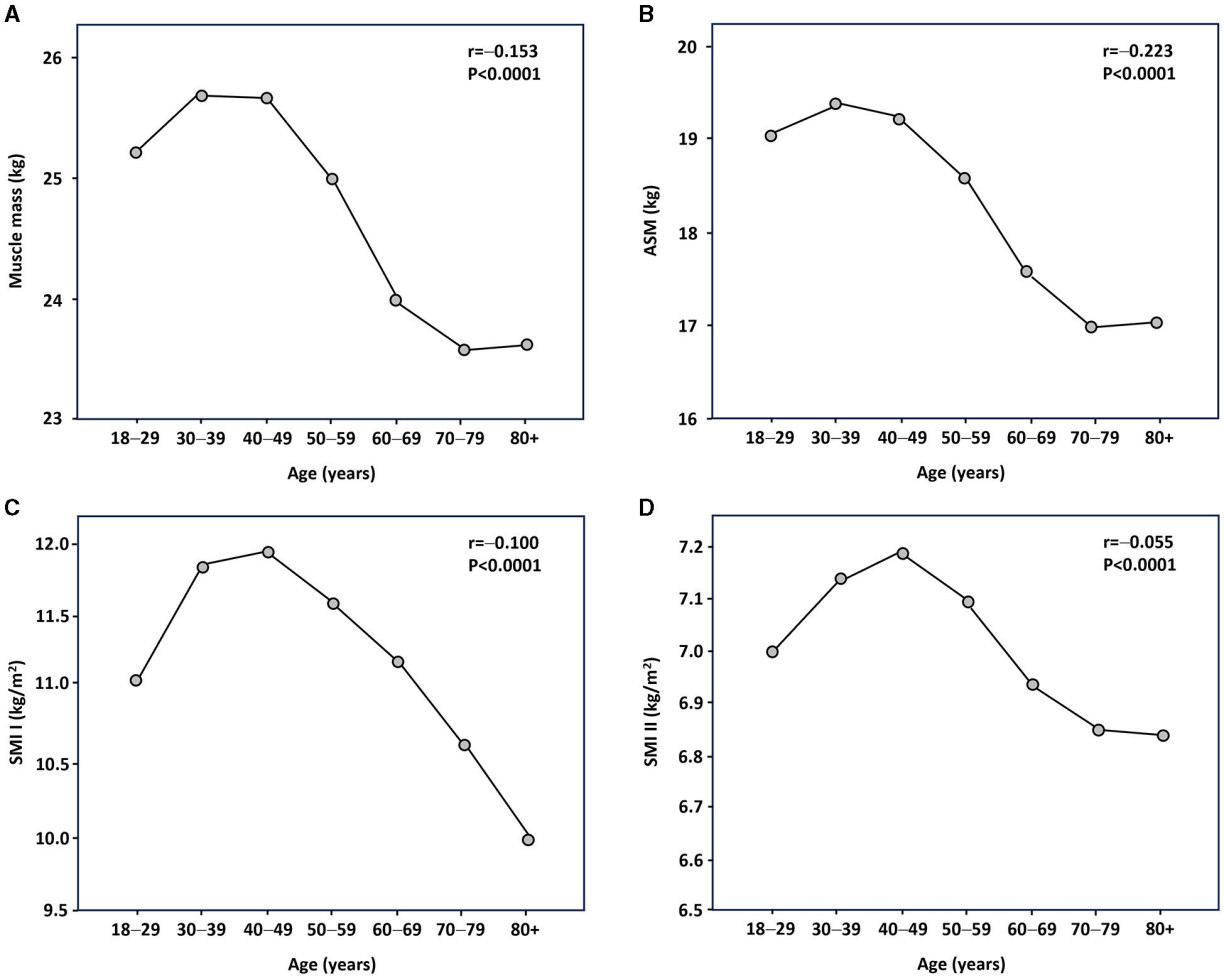
Relationship between age and muscle mass parameters in male participants as assessed by Pearson's statistics. **(A)** Muscle mass; **(B)** appendicular skeletal muscle (ASM); **(C)** skeletal muscle index (SMI) I; and **(D)** SMI II.

Figures 1, 2 show Pearson's correlation results for the association between age and muscle mass parameters in women and men, respectively. Muscle mass, ASM, and SMI II were inversely correlated with age, regardless of sex. SMI was positively associated with age in women, whereas inverse associations were noted in men. After adjusting the analysis for BMI, the relationship between age and SMI I in women became inverse and significant. No other significant changes were observed.

### 3.4 Normative values for muscle mass parameters

Normative values for muscle mass parameters in women and men, stratified by age groups, are listed in Tables 3, 4, respectively.

**Table 3:** Normative values for muscle mass parameters in women, stratified by age groups.

**Age groups (years)**	**Observations (*n*)**	**Centiles**					**Mean (SD)**
		**5th**	**25th**	**50th**	**75th**	**95th**	
**Muscle mass (kg)**							
18–29	1,544	21.4	23.1	24.6	26.9	30.9	25.2 (3.0)
30–39	2,452	21.6	23.2	25.0	27.5	31.9	25.7 (3.3)
40–49	3,101	21.4	23.4	25.2	27.5	31.3	25.6 (3.2)
50–59	1,878	21.1	22.8	24.6	26.7	30.5	25.0 (3.1)
60–69	1,127	20.7	22.3	23.5	25.0	29.0	23.9 (2.7)
70–79	368	20.4	21.8	23.0	24.6	29.0	23.5 (2.9)
80+	102	20.1	21.6	22.9	25.7	28.3	23.6 (2.6)
**Appendicular muscle mass (kg)**							
18–29	1,544	8.1	8.6	9.1	9	11.1	19.0 (2.4)
30–39	2,452	8.2	8.7	9.3	10.0	11.2	19.3 (2.7)
40–49	3,101	8.3	8.9	9.4	10.1	11.2	19.2 (2.5)
50–59	1,878	8.3	8.9	9.4	10.0	11.1	18.5 (2.4)
60–69	1,127	8.3	8.9	9.3	9.8	11.0	17.5 (2.2)
70–79	368	8.2	8.8	9.3	9.8	11.5	16.9 (2.0)
80+	102	8.1	8.9	9.3	10.0	11.0	17.0 (2.3)
**SMI I (kg/m** ^2^ **)**							
18–29	1,544	15.8	17.3	18.5	20.4	23.5	9.2 (0.9)
30–39	2,452	15.9	17.4	18.9	20.8	24.4	9.4 (0.9)
40–49	3,101	15.6	17.3	18.9	20.7	23.6	9.5 (0.9)
50–59	1,878	15.2	16.7	18.3	19.9	23.1	9.5 (0.9)
60–69	1,127	14.7	16.2	17.1	18.4	21.5	9.4 (0.8)
70–79	368	14.1	15.5	16.6	18.0	20.9	9.5 (1.2)
80+	102	13.4	15.4	16.5	18.9	20.7	9.5 (0.9)
**SMI II (kg/m** ^2^ **)**							
18–29	1,544	6.1	6.5	6.8	7.3	8.2	7.0 (0.6)
30–39	2,452	6.2	6.6	7.0	7.5	8.4	7.1 (0.7)
40–49	3,101	6.3	6.7	7.0	7.5	8.3	7.1 (0.6)
50–59	1,878	6.2	6.6	6.9	7.4	8.2	7.0 (0.6)
60–69	1,127	6.1	6.4	6.8	7.2	8.2	6.9 (0.6)
70–79	368	6.0	6.3	6.7	7.0	8.1	6.8 (0.7)
80+	102	5.8	6.3	6.7	7.1	8.2	6.8 (0.7)

**Table 4:** Normative values for muscle mass parameters in men, stratified by age groups.

**Age groups (years)**	**Observations (*n*)**	**Centiles**					**Mean (SD)**
		**5th**	**25th**	**50th**	**75th**	**95th**	
**Muscle mass (kg)**							
18–29	1,793	26.4	31.2	34.7	38.0	43.4	34.7 (5.2)
30–39	1,885	29.5	33.5	36.8	40.5	47.0	37.2 (5.4)
40–49	1,911	29.5	33.8	36.8	40.6	46.5	37.3 (5.2)
50–59	1,077	27.7	31.5	35.0	38.6	43.5	35.2 (4.8)
60–69	755	25.5	30.1	33.3	36.6	41.1	33.3 (4.9)
70–79	410	24.4	27.5	30.5	33.2	38.9	30.6 (4.2)
80+	222	22.3	25.0	26.5	30.0	35.0	27.6 (3.8)
**Appendicular muscle mass (kg)**							
18–29	1,793	9.0	10.1	11.0	11.9	13.2	26.1 (3.8)
30–39	1,885	10.0	11.0	11.8	12.5	14.0	27.6 (3.9)
40–49	1,911	10.0	11.1	11.9	12.7	14.0	27.6 (3.7)
50–59	1,077	9.8	10.8	11.5	12.3	13.4	26.2 (3.7)
60–69	755	9.3	10.4	11.1	11.8	13.2	24.8 (3.8)
70–79	410	9.0	9.8	10.6	11.3	12.2	22.7 (3.3)
80+	222	8.4	9.2	9.8	10.5	12.0	20.4 (3.0)
**SMI I (kg/m** ^2^ **)**							
18–29	1,793	20.2	23.6	26.0	28.3	32.5	11.0 (1.2)
30–39	1,885	21.8	24.8	27.3	29.9	34.7	11.8 (1.1)
40–49	1,911	21.9	25.1	27.3	30.1	34.1	11.9 (1.2)
50–59	1,077	20.3	23.4	25.9	28.8	32.2	11.5 (1.1)
60–69	755	18.3	22.3	24.8	27.3	31.1	11.2 (1.1)
70–79	410	17.8	20.2	22.7	24.7	29.1	10.6 (1.0)
80+	222	15.8	18.3	19.9	22.3	26.0	10.0 (1.0)
**SMI II (kg/m** ^2^ **)**							
18–29	1,793	6.9	7.7	8.3	8.8	9.6	8.3 (0.8)
30–39	1,885	7.5	8.2	8.7	9.2	10.1	8.8 (0.7)
40–49	1,911	7.6	8.3	8.8	9.3	10.2	8.8 (0.8)
50–59	1,077	7.3	8.0	8.6	9.1	9.9	8.6 (0.7)
60–69	755	6.8	7.8	8.3	8.8	9.7	8.3 (0.8)
70–79	410	6.7	7.3	7.9	8.4	9.1	7.8 (0.7)
80+	222	6.0	6.9	7.2	7.8	8.9	7.3 (0.7)

### 3.5 Reference percentiles for muscle mass parameters

**Figure 3:**
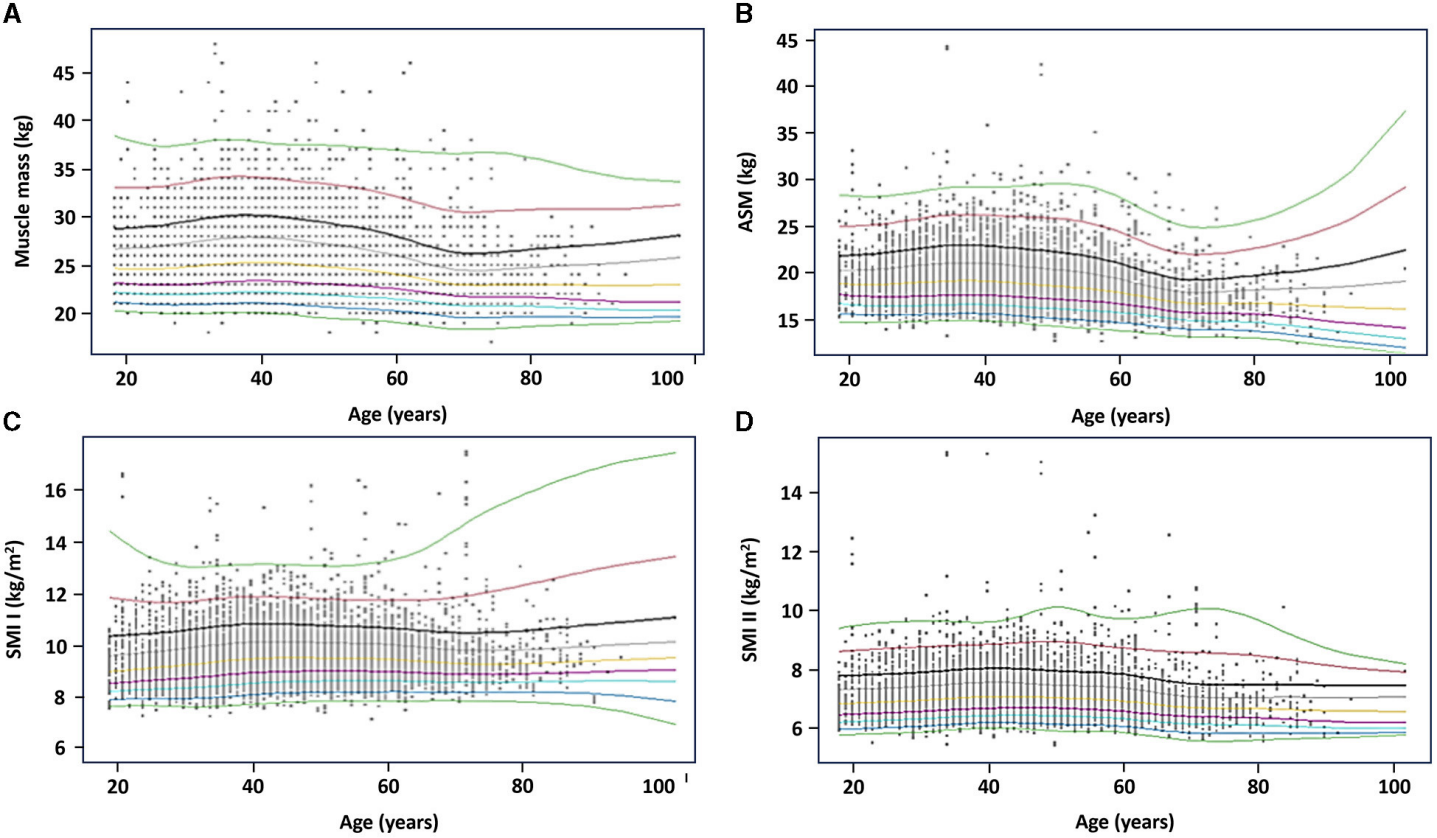
Reference percentiles for muscle mass parameters in women. **(A)** Muscle mass; **(B)** appendicular skeletal muscle (ASM); **(C)** skeletal muscle index (SMI) I; and **(D)** SMI II. The 5th, 25th, 50th, 75th, and 95th percentiles are depicted in black, red, green, light blue, and purple, respectively.

**Figure 4:**
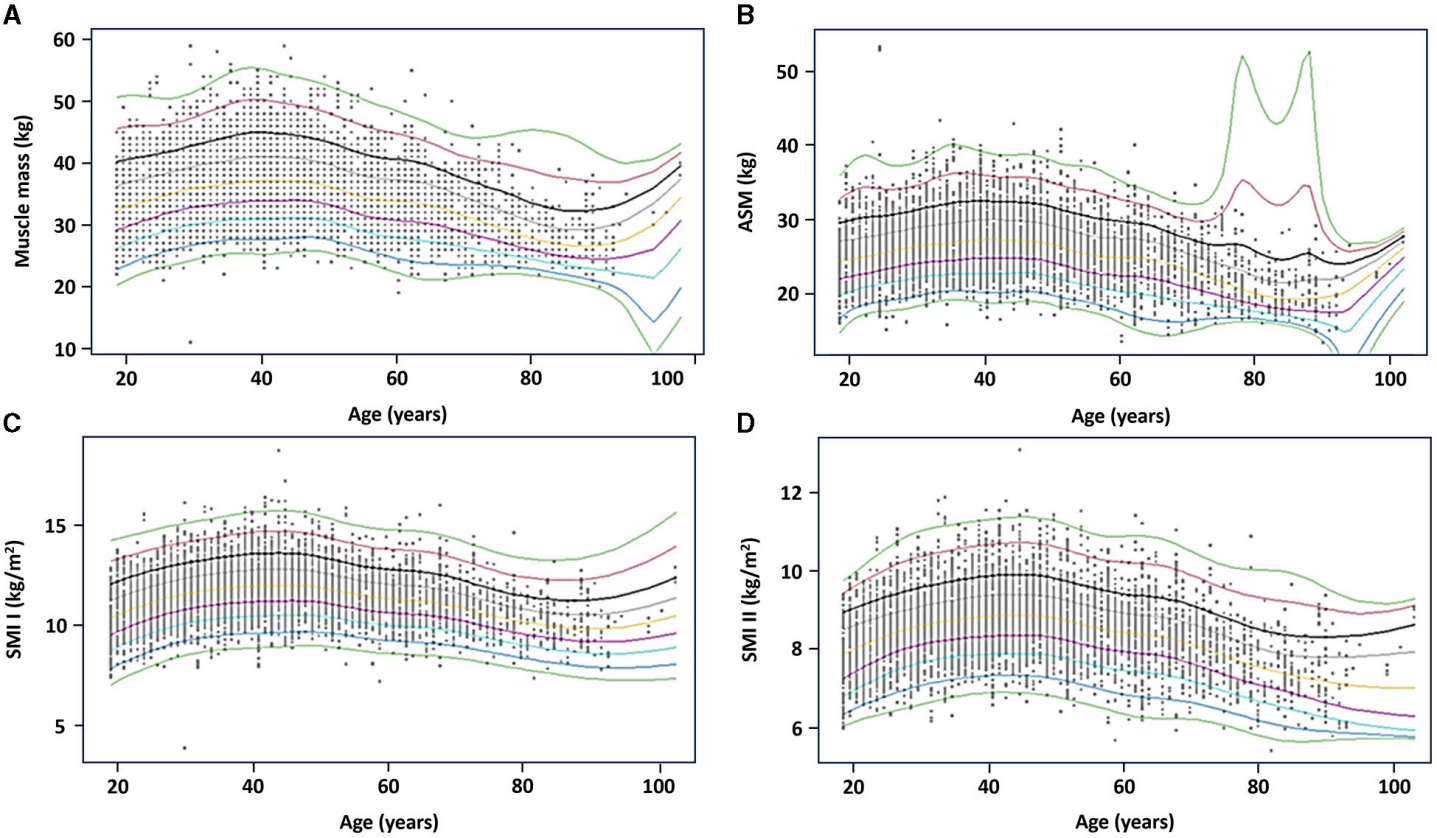
Reference percentiles for muscle mass parameters in men. **(A)** Muscle mass; **(B)** appendicular skeletal muscle (ASM); **(C)** skeletal muscle index (SMI) I; and **(D)** SMI II. The 5th, 25th, 50th, 75th, and 95th percentiles are depicted in black, red, green, light blue, and purple, respectively.

Reference percentiles for muscle mass, ASM, SMI I and II are also depicted as charts in Figures 3, 4 to facilitate their practical implementation.

## 4 Discussion

The present study was conducted to provide normative values and examine age- and sex-related changes in muscle mass parameters in a comprehensive sample of Brazilian adults. Such data might contribute to public health programs that aim to identify people at risk of negative events. Results of this study also provide cutoffs values to be used in clinical trials that aim to examine the effectiveness of strategies to improve muscle mass. In women, muscle mass reached a peak at 40–49 years and declined significantly from the sixth decade of life at a mean rate of 5.7% per decade. ASM reached a peak in the third decade of life and started to decline in those 50–59 years. In men, absolute and ASM reached a peak at 40–49 years and declined from the sixth decade of life. The mean decline rate during old age was slightly greater for ASM than muscle mass.

Most previous studies have examined age-related changes in fat-free mass (FMM). Kyle et al. (17) observed that FMM reached a peak at 35–44 years in men and at 45–54 years in women. Li et al. (16) suggested that the highest FFM values would be reached at 35–40 years in both sexes, with significant reductions starting approximately from the fifth decade of life. Schutz et al. (15) observed that FFM was significantly reduced in people >75 years. Regarding muscle mass, Janssen et al. (8) noted a curvilinear relationship between age and MRI-measured muscle mass in North American men and women, with significant declines from the fourth decade of life, mainly in the lower limbs. Similarly, Lee et al. (14) noted that BIA-muscle mass declined after 40 years in men. In contrast, muscle mass was more stable across life in women and only declined significantly in those over 55 years in the highest percentile.

Age-related changes in ASM have also been examined. Seino et al. (25) reported greater losses in ASM than in muscle mass in Japanese older adults. A relatively higher decline was noted in women (25). On the other hand, Makizako et al. (26) observed greater BIA-ASM losses in men, after examining more than 10,000 community-dwelling Japanese older adults. Clarck et al. (27) described that lean body mass and ASM decreased from 50 to 59 years in males and females.

Differences among studies might be explained by assessment tools, muscle mass parameters, and sample characteristics. Significant correlations have been observed between BIA and gold-standard assessment tools, such as MRI (7) and DEXA (13). However, the validity of BIA depends on numerous aspects, such as age, sex, hydration levels, pharmacological treatment, alcohol consumption, and the practice of exercise (28). Furthermore, age-related decline in FFM might be an overestimated measure of muscle atrophy, given that it involves changes in tissues other than muscle, including skin, bone, and fluids (28).

In the present study, women had an earlier and greater decline in ASM than in muscle mass, whereas similar decline rates were observed in men. These findings suggest that age-related changes in muscle mass aspects in Brazilian people might be sex-dependent. These differences between sexes could be due to the amount and intensity of physical activity (29), given that women spend more time on domestic work, whereas men are more active in recreational and occupational tasks (30, 31).

Domestic work commonly comprises repetitive physical tasks that combine the simultaneous utilization of both lower and upper limbs at low intensities (30, 31), thereby recruiting type I muscle fibers (32). These fibers are more resistant to fatigue but have a limited capacity to produce tension and increase their size in comparison to type II muscle fibers (32). In addition, domestic work might be associated with body pain (33), reducing physical activity levels, and contributing to the development of disuse atrophy (34). On the other hand, time spent by men in recreational activities might include the practice of exercise training and sports that stimulate muscle hypertrophy in the trunk and limbs (35).

Notably, the decline in muscle mass measures started at ~50 years of life and reaches the greatest losses during old age. Reductions rates of 20 and 21.4%, and 6.3 and 10.5%, were observed for absolute and ASM in men and women, respectively. These findings are supported by other studies that observed similar results (8, 17, 27). Such a scenario suggests that strategies to maintain muscle mass, especially of lower limbs, should be implemented during middle-age or urgently during the sixth and seventh decades of life. Potential therapeutic tools include physical exercise (36) and nutrition (37, 38).

Our findings provide normative values for muscle mass measures in Brazilian people. Absolute and ASM, and SMI II, were negatively and significantly associated with age in both men and women. SMI I was also inversely correlated with age in men, whereas it demonstrated a positive association in women, before adjusted the analysis for BMI. These results might indicate that adjust muscle mass measures for squared height is not the best method to create SMI in this population.

The best adjustment variable to be used in sarcopenia diagnosis is still under debate, and the revised European consensus on sarcopenia mentions that height and BMI are accepted variables (10). However, both measures might be affected by nutrition and physical exercise. This aspect deserves attention because our sample was composed of people who attend a private nutrition clinic and received individual accompaniment by certified nutritionists. People who look for nutritional counseling usually also adhere to other healthy behaviors, such as physical exercise, in an attempt to accelerate or maximize nutritional benefits. Hence, future studies examining other adjusting parameters less affected by health habits are still needed.

The present study is not free of limitations. First, our results should be carefully extrapolated to people in other conditions. Second, although all patients were advised to attend to the clinic under standard conditions, the possibility that some of them did not follow recommendations cannot be ruled out. Third, evidence has suggested that normative values for muscle mass are better presented by categorizing patients according to BMI (39). However, BIA loses sensibility in people with high BMI values (28, 40). Fourth, the lack of information about participants' physical activity levels, exercise adherence, nutritional aims, the prevalence of diseases, and pharmacological therapy impeded us to provide a better characterization of the study population. Fifth, normative values provided in the present study still need to be tested across health parameters and negative events. Sixth, assessments occurred at different times of the day. Finally, the cross-sectional design of the study does not allow any inference to be drawn on the time course of changes in the variables considered and on cause-effect relationships.

In conclusion, the present study provided normative values for absolute and relative muscle mass and ASM in Brazilian adults. Furthermore, important specific age-related changes in muscle mass parameters were observed. Specifically, women experienced a gradual decline in absolute muscle mass from the age of 60, whereas an earlier decline, from the age of 50, was noted in ASM. On the other hand, absolute and ASM were similarly reduced from the sixth decade of life in men. These data have public health implications and might serve as reference tables to guide health professionals.

## Data availability statement

The raw data supporting the conclusions of this article will be made available by the authors, without undue reservation.

## Ethics statement

The studies involving humans were approved by the Ethics Committee of University Centre UDF (protocol #: 5.975.561). The studies were conducted in accordance with the local legislation and institutional requirements. The participants provided their written informed consent to participate in this study.

## Author contributions

HJC-J: Conceptualization, Data curation, Formal analysis, Investigation, Methodology, Writing – original draft, Writing – review & editing. FLM: Conceptualization, Data curation, Formal analysis, Funding acquisition, Investigation, Methodology, Writing – original draft, Writing – review & editing. CVS: Conceptualization, Data curation, Formal analysis, Investigation, Methodology, Writing – original draft, Writing – review & editing. SdSA: Conceptualization, Data curation, Formal analysis, Funding acquisition, Investigation, Methodology, Supervision, Writing – original draft, Writing – review & editing. EM: Conceptualization, Data curation, Formal analysis, Funding acquisition, Investigation, Methodology, Writing – original draft, Writing – review & editing.
